# Pulsatile Tinnitus: A Comprehensive Clinical Approach to Diagnosis and Management

**DOI:** 10.3390/jcm14134428

**Published:** 2025-06-22

**Authors:** Sofía Pacheco-López, Jose Pablo Martínez-Barbero, Heriberto Busquier-Hernández, Juan García-Valdecasas-Bernal, Juan Manuel Espinosa-Sánchez

**Affiliations:** 1 Division of Otology, Audiology and Lateral Skull Base Surgery, Department of Otolaryngology, Hospital Universitario Virgen de las Nieves, 18014 Granada, Spain; 2 Division of Neuroradiology, Department of Radiology, Hospital Universitario Virgen de las Nieves, 18014 Granada, Spain; 3 TECe22-Imagen Médica Avanzada, Instituto de Investigación Biosanitaria ibs.GRANADA, 18012 Granada, Spain; 4 Division of Otoneurology, Department of Otolaryngology, Hospital Universitario Virgen de las Nieves, 18014 Granada, Spain; 5 Otology and Neurotology Group CTS495, Instituto de Investigación Biosanitaria ibs.GRANADA, 18012 Granada, Spain; 6 Division of Otolaryngology, Department of Surgery, Universidad de Granada, 18016 Granada, Spain; 7 Sensorineural Pathology Programme, Centro de Investigación Biomédica en Red en Enfermedades Raras (CIBERER), 28029 Madrid, Spain

**Keywords:** tinnitus, pulsatile tinnitus, clinical approach, arteriovenous fistula, idiopathic intracranial hypertension

## Abstract

Pulsatile tinnitus (PT) is a subtype of tinnitus characterized by a perception of heartbeat-synchronous sound. It represents approximately 5–10% of all tinnitus cases and may have either a vascular or non-vascular etiology. Accurate diagnosis is crucial due to the potentially serious implications this condition can entail. Assessment through anamnesis and physical examination may often suggest a diagnosis of PT, but it is rarely definitive. Therefore, a comprehensive and specific imaging diagnostic protocol is essential when evaluating PT. A lack of consensus has been identified regarding the use of a standardized protocol for both pulsatile and non-pulsatile tinnitus, whether unilateral or bilateral. Consequently, neuroradiologists, otologists, and otoneurologists from a tertiary hospital have developed a new imaging diagnostic protocol for PT. The aim of this article is to present an updated approach to the diagnostic and therapeutic management of PT, aiming to establish a protocol that serves as a guide for clinicians assessing this symptom. In patients with bilateral PT, systemic conditions leading to increased cardiac output should generally be ruled out; in unilateral cases, focused imaging studies should be performed to exclude organic etiologies at the cervical and cranial levels.

## 1. Introduction

Tinnitus is defined as the perception of sound without an external triggering source, meaning that it cannot be heard by others nearby and can significantly affect patient’s quality of life [1,2,3,4,5,6,7,8,9]. Traditionally, tinnitus has been categorized into subjective (90–95% of cases), where the sound is perceptible only to the patient, and objective (5–10%), where the sound may originate from a detectable somatic source and, in some instances, may even be audible to an external observer [10]. Nevertheless, the term “objective tinnitus” is increasingly discouraged and the term “somatosound” is now preferred when referring to tinnitus with a demonstrable bodily origin, including cases related to vascular, muscular, or osseous anomalies, that may or may not be detectable through imaging [11,12]. Pulsatile tinnitus (PT) is a subtype of somatosound characterized by a rhythmic perception of sound that is synchronous with the patient’s heartbeat. This sets it apart from non-pulsatile tinnitus, which is usually continuous and more commonly associated with sensorineural hearing loss. While the vast majority of tinnitus cases are subjective and non-pulsatile, PT often reflects an underlying somatic or vascular origin and may be detectable through imaging techniques. Another classification system categorizes tinnitus as primary, when it is idiopathic (with or without associated sensorineural hearing loss), or secondary, when there is an underlying etiology distinct from sensorineural hearing loss or another identifiable organic condition [13].

PT presents a diagnostic challenge due to its wide range of potential etiologies, including vascular anomalies, neoplasms, and bone disorders. The diagnostic approach to PT differs substantially from that of non-pulsatile tinnitus. For instance, bilateral non-pulsatile tinnitus with symmetrical hearing loss is typically associated with damage to outer hair cells in the cochlea and generally does not require neuroimaging, as imaging findings are usually unremarkable in such cases [8,13]. In contrast, in patients with unilateral non-pulsatile tinnitus, magnetic resonance imaging (MRI) of the internal auditory canal (IAC) and cerebellopontine angle (CPA) should be requested to rule out pathologies such as vestibular schwannoma [14,15,16,17,18,19,20]. However, PT often requires imaging evaluation to identify underlying potentially treatable causes, especially when symptoms are unilateral or associated with additional findings, and in cases in which the clinical history and physical examination do not suggest an obvious cause. Despite its clinical relevance, there is currently no consensus on standardized imaging protocols for patients with PT, and there is an ongoing debate as to whether all patients with PT should undergo imaging or whether this decision should be guided by clinical examination and history alone.

Given the complexity and diagnostic uncertainty surrounding PT, the aim of this article is to provide a comprehensive and structured clinical framework for the assessment and management of PT. By reviewing the available literature and incorporating expert consensus from a multidisciplinary team—including neuroradiologists and otolaryngologists specialized in otology and otoneurology at Hospital Universitario Virgen de las Nieves (Granada, Spain)—this study seeks to address the current gaps in clinical decision-making and clarify the role of imaging in PT evaluation.

## 2. Material and Methods

This scoping review was conducted following the guidelines from the Preferred Re-porting Items for Systematic Review and Meta-Analysis extension for Scoping Reviews (PRISMA-ScR) [21]. A comprehensive search strategy was conducted using the PubMed, Embase, and ScienceDirect databases. We used a combination of Medical Subject Headings (MeSH) and free-test terms related to pulsatile tinnitus, its etiology, diagnosis, and clinical approach. In these terms, “objective tinnitus” was not included because it was not considered to be the appropriate term. However, the authors consider that the terms that were included covered the required concept. For all eligible articles, data extraction was performed, and basic information was extracted. A data-charting form was collaboratively developed by two reviewers to identify the variables to be extracted. The reviewers independently charted the data, discussed their findings, and iteratively refined the data-charting form throughout the process. 

The inclusion criteria were publications from the last 30 years on adherence to pulsatile tinnitus and clinical approach, which were written in English or Spanish. The most recent search was conducted on 30th October 2024. The initial selection of the articles was based on the title and abstract of the articles, followed by a full-text assessment.

All articles not related to pulsatile tinnitus and its clinical approach were excluded. Publications focusing on non-pulsatile tinnitus, as well as case reports, case series, commentaries, notes, letters, and editorials were also excluded. Additionally, studies published more than 30 years ago or written in languages other than English or Spanish were not included in this scoping review.

The complete search strategy for Pubmed is as follows: (“pulsatile tinnitus”[tiab] OR “vascular tinnitus”[tiab] OR ”somatosound”[tiab]) AND (“diagnosis”[tiab] OR “clinical evaluation”[tiab] OR “diagnostic approach”[tiab] OR “clinical management”[tiab] OR “therapeutic approach”[tiab] OR “treatment”[tiab]). Similar terms were adapted for the other databases.

**Figure 1:**
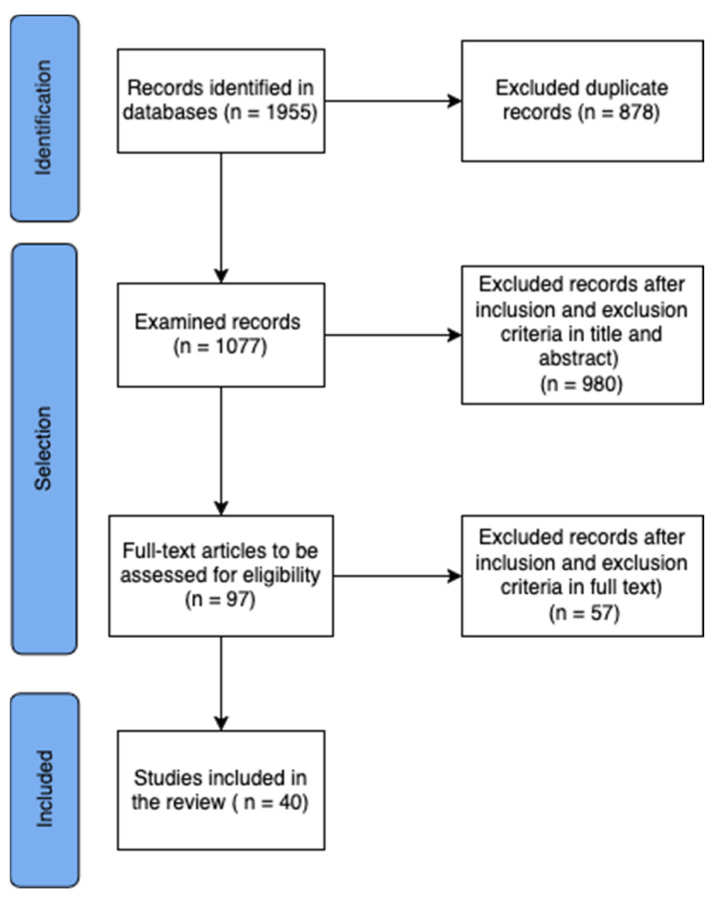
The PRISMA—ScR flow diagram.

Out of the initial 1955 articles identified through the search, 40 studies were selected to the review (Figure 1).

All relevant outcome data extracted from each source of evidence are presented in Supplementary Table S1, in accordance with PRISMA-ScR.

## 3. Pulsatile Tinnitus

PT necessitates careful attention and thorough examination to achieve an accurate etiological diagnosis due to the potential severe implications it may carry. PT is synchronous with the patient’s pulse and may result from an acquired or congenital vascular anomaly, producing a sound that, in some instances, may even be audible to the examiner [1,3,4,5,9,10,22]. It is important to differentiate PT from rhythmic tinnitus, which is not synchronous with the pulse and is caused by somatosounds generated by muscle clicks, the perception of respiratory sounds, joint clicks, or other similar mechanisms; and may be termed “pseudo-pulsatile” tinnitus [12].

Regarding its pathophysiology, all PT results from the perception of nearby pulsatile flow by the cochlea, where the sensory cells are stimulated by the sound of this pulsatile flow. It is necessary to note that all intracranial fluids are pulsatile, not only arterial blood but also venous blood and cerebrospinal fluid (CSF), and, consequently, any of these can be responsible for causing PT [12,23]. In this context, two main mechanisms are identified as causes of PT:

- The presence of turbulence in a vessel near the inner ear, which occurs when a vascular compartment experiences accelerated flow. This is typically observed distal to a vascular stenosis or arteriovenous malformation, or because of increased blood flow.
- Amplification of the sound of normal blood flow at the base of the skull. This can happen under the following conditions:
  ○ Dehiscence or loss of the bony covering that isolates the inner ear from intracranial fluid.
  ○ Conductive hearing loss due to reduced sound masking or altered sound conduction. In such cases, the inner ear retains excellent hearing, allowing effective perception of auditory stimuli via bone conduction, while the masking effect of ambient sounds is reduced due to impaired air conduction of external sounds. As a result, any intracranial sound, such as the pulse of arteries near the ear, will be transmitted through the bone and perceived by the inner ear via bone conduction. Hence, any form of conductive hearing loss (such as earwax blockage, chronic otitis media, otosclerosis, or otitis media with effusion) can lead to PT [10,24].

Thus, it is understood that imaging studies for PT should focus on evaluating intracranial vasculature and the temporal bone, where the inner ear is located [24,25].

### 3.1. Diagnostic Assessment

In the diagnostic evaluation of PT, it is essential to emphasize a detailed clinical history. First, it is important to confirm that the sound is synchronous with the heartbeat and increases with physical activity, as these features typically indicate a vascular etiology. In contrast, asynchronous PT is often related to a mechanical cause, such as palatal or middle ear myoclonus. The onset pattern of PT is also informative: a sudden onset may suggest conditions like hypertensive crises, vascular anomalies (e.g., aneurysms, fistulas, carotid dissections), or vascular compression by a cervical mass. Insidious onset is more common in cases of idiopathic intracranial hypertension (IIH) or anemia. The sound tone can provide further diagnostic clues. A low-frequency sound (e.g., a “buzz” or “murmur”) is often venous in origin, while a high-frequency sound (e.g., a “whistle” or “hiss”) is typically arterial [26]. Past medical history is another critical element, as conditions such as hypertension, hyperthyroidism, anemia, pregnancy, and medication changes (e.g., enalapril or verapamil [27]) may contribute to PT. Other factors, such as traumatic brain injury (TBI), may suggest an arteriovenous fistula, weight gain could point to IIH, and recent cervical manipulation might indicate arterial dissection. Family history should also be considered, particularly regarding vascular malformations or otosclerosis. Associated symptoms are key in narrowing down the diagnosis. Auditory symptoms, such as hearing loss or ear fullness, may suggest conductive hearing loss from otitis media with effusion, a middle ear mass, or an inner ear disorder. Autophony, ear fullness, or sound-induced vertigo may indicate superior canal dehiscence syndrome (SCDS). Headaches, particularly hemicranial, or visual disturbances could suggest a migraine with brainstem aura (which can trigger PT during the aura phase or due to vasodilation during the headache) [28] or IIH [22]. Finally, it is essential to assess the severity of PT, including factors that exacerbate or relieve the condition, as well as the impact on the patient’s quality of life, to guide appropriate management.

Second, a thorough physical examination is crucial in the evaluation of PT. Otoscopy should be performed to rule out common causes of conductive hearing loss associated with PT, such as otitis media with effusion, chronic otitis media, acute otitis media, or the presence of a retrotympanic mass (Table 1). Additionally, auscultation and palpation of the neck, chest, and skull are essential to assess for the presence of bruits at the cardiac level (in cases of arrhythmias or heart disease), cervical region (in carotid disease such as aneurysms or atherosclerosis), or bruits in the temporal, orbital, or retroauricular regions (in arteriovenous fistulas or malformations). Furthermore, to differentiate between venous and arterial causes of PT, certain cervical maneuvers can be helpful. These include compressing the internal jugular vein or the carotid artery, performing a Valsalva maneuver, or rotating the head, all while observing if the PT is altered. Venous PT tends to be low-pitched and often improves or disappears with compression of the ipsilateral jugular vein, head rotation toward the healthy side, or performing the Valsalva maneuver. If PT worsens with jugular compression, it could indicate ipsilateral condylar venous overflow (as blood flow is diverted to dilated condylar veins) or arterial involvement (where narrowing of the artery without occlusion leads to increased blood flow velocity) [12,22]. Arterial PT is typically synchronous with the arterial pulse and may not improve or disappear with jugular compression. Other factors to consider during the examination include assessing blood pressure and body mass index (BMI), as atherosclerosis is more likely in elderly, hypertensive, or overweight patients and IIH more frequently affects women with overweight [22]. Ophthalmological evaluation is essential if IIH is suspected, as this condition often presents with papilledema. Additionally, other areas such as the temporomandibular joint, cranial nerves, and vestibular function may need to be evaluated to exclude other potential causes of PT.

Complementary tests should be selected based on the findings from the medical history and physical examination. Audiological evaluations such as pure-tone audiometry and tympanometry with stapedial reflex testing can be useful to determine whether the patient has conductive or sensorineural hearing loss. Additionally, speech audiometry may provide further insight in certain cases, as part of a complete audiological evaluation to detect sensorineural vs. conductive hearing loss and its functional repercussions (speech intelligibility). Additionally, the rollover phenomenon, defined as a decline in speech recognition performance at suprathreshold intensities following a previously achieved maximum score, may suggest the presence of a retrocochlear lesion, which can be a potential cause of PT (this phenomenon is quantified using the rollover index). In some patients, the PT itself may mask certain frequencies due to its low intensity, even if there is no true hearing loss. In these cases, performing audiometry in a posture where the tinnitus is not perceived, or using otoacoustic emissions or auditory brainstem response, might be useful. In some situations, for instance bilateral PT, laboratory tests may be necessary, including complete blood count and thyroid function tests, to rule out conditions that may cause PT, such as anemia, thrombocythemia, or hyperthyroidism. Additionally, a lumbar puncture is indicated in cases of suspected IIH as its diagnosis required confirmation of an elevation of CSF pressure [22,24]. In some patients, conventional peri-auricular and neck auscultation may not objectively detect the presence of a PT; for this reason the use of transcanal sound recordings as a screening tool may prevent patients from experiencing the unnecessary risks of DSA in the diagnostic work-up. The absence of a pulsatile sound detected by transcanal sound recordings excludes a dAVF (100% sensitivity) [29,30]

If no etiological diagnosis has been made thus far, imaging studies should be considered, which is the focus of the present document. Imaging plays a crucial role in the diagnosis of PT. The imaging strategy should be guided by the patient’s clinical history and physical examination, including otoscopic findings or the presence of an audible bruit. In patients with continuous PT and normal otoscopic examination, contrast-enhanced brain MRI is the preferred imaging study. MRI generally has the highest sensitivity for detecting the more dangerous potential causes of PT, such as dural arteriovenous fistulas (dAVF), arterial disease, IIH, and neoplasms, and it does not expose the patient to ionizing radiation. MRI protocol should include, in addition to diffusion-weighted sequences, T2-weighted FLAIR sequences, T1-weighted pre- and post-contrast 3D images, and high-resolution heavily weighted T2 images (such as FIESTA, Drive or Ballance, depending on vendors). Additionally, MRI allows for arterial or venous MR angiography (MRA) with time-of-flight (TOF) or phase contrast sequences, arterial spin labeling non-contrast perfusion (ASL), and time-resolved MRA sequences to assess vascular structures [24]. If MRI is normal, or in patients with conductive hearing loss and normal otoscopy, or when a retrotympanic mass is visible on otoscopic examination, a high-resolution Computed Tomography (CT) of the head and temporal bone with contrast should be considered to rule out other pathologies such as otosclerosis, SCDS, aberrant internal carotid artery (ICA), jugular bulb abnormalities, sigmoid sinus anomalies, and other structural abnormalities of the auditory system. For patients with contraindications to MRI (e.g., metallic implants, claustrophobia), CT angiography (CTA) is sometimes used as an alternative. If cervical vascular pathology is suspected, a CTA of the supra-aortic trunks (SATs) in conjunction with a head CT may be considered. However, cervical Doppler ultrasound is not generally recommended, as cervical vascular involvement is uncommon in PT cases. Lastly, digital subtraction angiography (DSA) is now generally reserved for situations where there is strong suspicion of an arterial cause or high likelihood of dAVF, and it can also be used as a therapeutic tool [12,24,25]. It is important to interpret imaging findings carefully, considering the clinical presentation. Certain anatomical variants, such as a high-riding jugular bulb, ICA dehiscence, neurovascular loops, or arteriosclerosis, may be incidentally found in asymptomatic patients. However, if one of these variants is identified ipsilateral to the PT, it could potentially explain the symptom, provided other pathologies have been excluded.

Finally, an appropriate psychological evaluation and assessment of tinnitus-related quality of life using questionnaires such as the Tinnitus Functional Index (TFI) (TFI) should be performed [31]. Occasionally, referral to a psychologist or psychiatrist may be necessary to address the mental health aspects of the condition. This holistic approach ensures both physical and emotional aspects of the patient’s well-being are considered, leading to comprehensive management.

### 3.2. Etiological Diagnosis

#### 3.2.1. Unilateral Pulsatile Tinnitus

In patients where conductive hearing loss has been ruled out, we should consider performing imaging studies to exclude a vascular etiology, such as turbulence in blood flow near the inner ear, which may cause PT. Based on this, we will classify PT as either vascular or non-vascular in origin, to guide further evaluation, as summarized in Table 2 [24,32].

##### Vascular Etiology

Arterial etiology

The main causes of PT of vascular etiology are described in Table 2.

A. Vascular stenosis

Stenosis of any vessel near the inner ear can lead to PT due to increased turbulent blood flow at the site of narrowing or compensatory increased flow in another cervical artery [34]. This stenosis can result from conditions such as atherosclerosis, fibromuscular dysplasia, or arterial dissection.

Atherosclerosis

**Figure 2:**
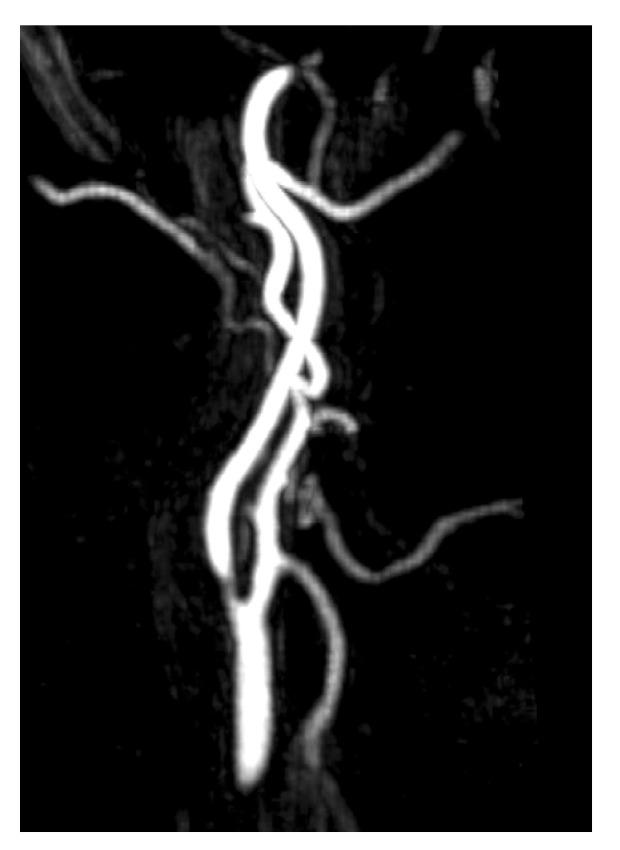
MRA imaging of vascular stenosis due to atherosclerosis in a 72-year-old female patient with cardiovascular risk factors. She presented with PT in the right ear, synchronous with her pulse. Compression of the right carotid artery reduced the perception of the tinnitus. Sagital-MIP MRA revealed atheromatous plaques in both carotid bifurcations, with no significant stenosis on the left side. However, on the right side, there was short segmental stenosis, with a caliber reduction of over 70%. Revascularization was performed by the angiology team, resulting in the resolution of the PT.

Atherosclerosis is one of the most common causes of arterial PT (Figure 2), especially in elderly patients with cardiovascular risk factors such as hypertension, diabetes, dyslipidemia, smoking, or a history of stroke or myocardial infarction. PT can occur when atherosclerotic plaque affects any part of the ICA, more frequently from the carotid bifurcation to the intracranial siphon, or the subclavian and vertebrobasilar arteries. Stenosis below the carotid bulb rarely causes PT, as the distance from the inner ear is too great to create perceptible turbulence (it is believed that the atherosclerotic plaque or stenosis must be above the level of C4) [22,34].

In patients with suspected atherosclerotic PT, neck auscultation may reveal a “murmur”-like bruit, which may change with head rotation or extension. Significant stenosis in a cervical vessel can be detected using CTA, MRA, or Doppler ultrasound [22], though the latter is not typically a first-line diagnostic test for arterial PT. Doppler ultrasound is not indicated because stenosis below the carotid bifurcation seldom causes PT, and for carotid stenosis to lead to PT, it must be significant (>70%) [12,34]. Given these considerations, MRI combined with MRA is the recommended imaging modality when vascular stenosis is suspected. In some cases, large cervical tumors may compress major cervical vessels, such as the carotid artery, resulting in stenosis and turbulent blood flow, which can cause PT. In such patients, a CT scan may be requested before an MRI to identify the cause of the vascular compression.

Treatment of atherosclerotic plaque (through medical management or revascularization) often leads to resolution of the PT. Effective management of the cardiovascular risk factors is critical to prevent progression [10,12].

Fibromuscular dysplasia

Fibromuscular dysplasia (FMD) is a non-atherosclerotic and non-inflammatory vascular disease primarily affecting medium-sized arteries and is most prevalent in middle-aged women (20–60 years old). The etiology of this condition is unknown, although it is believed to have a genetic component. It is a multifocal disease, most commonly affecting the renal arteries (in 60–75% of patients) and cervical arteries (up to 25%), but it can also involve the cervical vertebral arteries and, less frequently, the intra-abdominal arteries (9%) or external iliac arteries (5%) [25]. Clinically, many patients are asymptomatic; however, some may experience PT, headaches, vertigo, or even stroke. PT occurs due to stenosis and dilatations in cervical vessels that generate turbulent flow perceived as somatosound. On imaging, FMD presents with the classic “string of beads” or “coin stack” appearance, with alternating areas of vascular narrowing and dilatation [35,36]. The diagnosis is typically made using MRA or CTA and confirmed through cerebral angiography. Treatment generally involves antiplatelet prophylaxis, although some patients may require angioplasty, stenting, or anticoagulation [10,12,34].

Arterial dissection

A rare cause of PT is cranio-cervical arterial dissection. Arterial dissection occurs when a tear in the intima (inner layer) of an artery allows blood to accumulate between layers of the vessel wall, forming a hematoma that narrows the lumen [12,32]. The parietal hematoma typically undergoes spontaneous resorption; however, the dissected segment can evolve in three ways: restoration of normal arterial caliber, persistence of a post-dissection scar stenosis, or the formation of a pseudoaneurysm [10]. This mechanism may lead to PT, although it is infrequent (accounting for 2–6% of cases), as the stenosis generates turbulent blood flow, either due to vessel narrowing from the hematoma or from the formation of a dissecting pseudoaneurysm [10,32]. In these patients, it is important to rule out an underlying connective tissue disorder, although this is present in a significant minority. Dissections can be spontaneous or traumatic, commonly affecting the cervical ICA and distal intracranial vertebral arteries (VAs) [10,12]. Though it is a rare cause of PT, it is a critical diagnosis due to the risk of ischemic events such as stroke. Symptoms associated with arterial dissections include headache, neck pain, facial pain, Horner’s syndrome, or even stroke if the dissection affects intracranial vessels. The diagnosis is established using MRA or CTA, where stenosis is often visualized below the carotid bulb or in the extravertebral segments of the VA [12]. Management generally includes anticoagulation or antiplatelet therapy to prevent thrombus formation and embolism. In cases of progressive stenosis or pseudoaneurysm formation, endovascular stenting may be required if the dissection is associated with hemodynamically significant progressive stenosis [10].

B. Variants of normal skull base anatomy

Aberrant and/or dehiscent ICA

**Figure 3:**
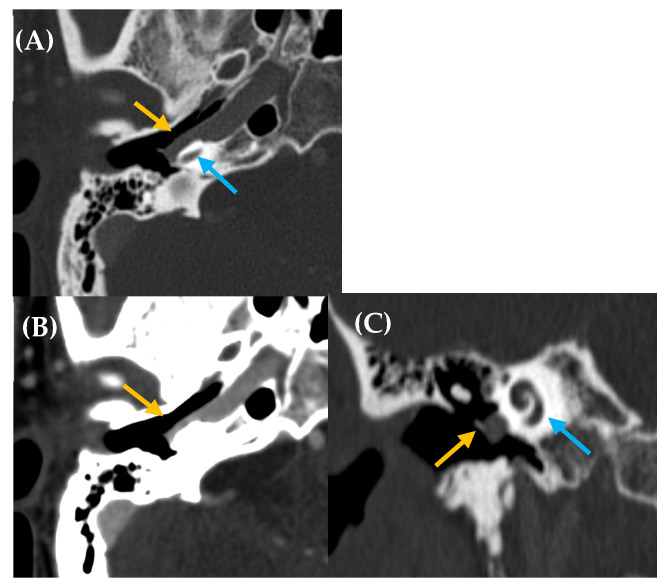
Aberrant intratympanic course of the right internal carotid artery, which is in contact with the cochlea (yellow arrow: aberrant ICA; blue arrow: cochlea). A 33-year-old man with right-sided PT of several years’ duration. Otoscopy revealed a pulsatile tumor in the right ear. CT imaging (**A**), axial plane bone window; (**B**), axial plane, contrast enhanced with soft tissue window; and (**C**), MPR coronal plane reconstruction) demonstrated a space-occupying lesion with a tubular appearance in the right mesotympanum, adjacent to the cochlear promontory, along with an enlarged inferior tympanic canaliculus and absence of the carotidotympanic bone septum.

Another potential cause of PT involves anatomical variants affecting the arteries at the base of the skull, with the aberrant ICA being the most common (Figure 3). In these patients, the ICA may take a different pathway, deviating from its normal anteromedial course through the petrous carotid canal to extend laterally into the middle ear. This variant is thought to arise when the cervical portion of the ICA either fails to develop properly or regresses. Consequently, the inferior tympanic and caroticotympanic arteries (two embryonic vessels) enlarge to compensate for the missing segment of the ICA [1,10]. The inferior tympanic artery typically extends laterally through the temporal bone into the middle ear, and in the case of an aberrant ICA, it follows a very similar course [1,37,38]. On otoscopy, a pulsatile vascular structure might sometimes be visible behind the tympanic membrane in cases of an aberrant ICA, which can occasionally be dehiscent. The carotid pulse is directly transmitted to the membranous labyrinth, leading to the perception of PT. It is crucial to conduct appropriate imaging studies (CT and MRA) to identify this variant, as it can sometimes be mistaken for a tympanic paraganglioma, a misdiagnosis with potentially catastrophic consequences during surgical intervention. If the ICA dehiscence is misdiagnosed and approached surgically, injury to the artery may occur and potentially result in massive hemorrhage, stroke, or even fatality [22].

We refer to dehiscent ICA when there is thinning or absence of the normal bony covering of the ICA, typically near the basal turn of the cochlea. This condition can be confirmed through imaging studies such as CT scans. The lack of bony separation allows for the direct transmission of the arterial pulse from the ICA to the membranous labyrinth, potentially causing PT and also appearing as a vascular mass behind the tympanic membrane. ICA dehiscence can occur with or without the presence of an aberrant ICA [1,10].

Persistent stapedial artery

The persistent stapedial artery (PSA) is an embryological vessel that originates from the second primitive aortic arch. It divides into a dorsal branch (the future middle meningeal artery) and a ventral branch (the future maxillary and mandibular arteries). These branches connect with branches from the external carotid artery (ECA) during the third fetal month, causing the regression of the stapedial artery. If the stapedial artery persists into postnatal life, the middle meningeal artery arising from it will be absent, and as a result, the foramen spinosum (which typically contains the middle meningeal artery) will also be absent on that side. In patients with a PSA, this artery supplies the usual vascular territory of the middle meningeal artery. On CT, it can be identified as a small artery originating from the petrous ICA and running into the hypotympanum through the obturator foramen, situated between the stapes crura, following the tympanic portion of the facial canal. Additionally, CT will reveal the absence of the foramen spinosum and the proximal portion of the middle meningeal artery. In Table 3, the main CT signs suggestive of a PSA are detailed. This artery can be a source of PT and can also present as a retrotympanic mass [1,10,37].

C. Arterial compression of vestibulocochlear nerve

The loops of the anterior inferior cerebellar artery (AICA) are often located very close to the cochleovestibular nerve or cranial nerve VIII (CN VIII) within the CPA and the IAC. In patients with this anatomical relationship, they typically present with a Meniere-like syndrome, characterized by fluctuating sensorineural hearing loss, vertigo, and tinnitus. Chavda and McDermott described a simple method for the classification of AICA loops (Table 4) [39], but an association between this classification and otoneurologic symptoms is not clearly present. When tinnitus arises from nerve compression, it tends to be continuous and non-pulsatile. However, if the artery contacts the bony wall of the IAC, it can generate PT due to the transmission of the arterial pulse through the cranial bone into the inner ear [10,22,37]. Nevertheless, caution must be exercised when interpreting these imaging findings and linking them to PT, as neurovascular loops are highly prevalent in asymptomatic individuals, with rates ranging from 14 to 34% [10,40,41]. Some studies have found significant differences in the frequency of contact between AICA loops and the CN VIII in symptomatic patients compared to control subjects, while others have not observed such differences. Therefore, this finding should be considered only after ruling out other potential causes of PT [10,37,40,42]. The diagnosis is often made using MRI, and auditory brainstem response (ABR) may show alterations in the nerve if compression is present (Table 5) [43,44]. In some cases, there may be a mass or cyst in the CPA causing the compression syndrome. Some authors report 77–79% improvement after performing vascular decompression of CN VIII; however, surgical intervention should be approached with caution. It is essential to rule out other potential causes of the patient’s symptoms before recommending surgery [45].

D. Cerebral aneurysms

Cerebral aneurysms arising from the ICA and vertebrobasilar territory are an uncommon cause of PT. These aneurysms can lead to turbulent blood flow within the affected artery, which may result in PT. However, PT is a rare manifestation of aneurysms affecting the cerebral–cervical vasculature [10,41]. In some cases, an aneurysm may compress the vestibulocochlear nerve, which could lead to the development of PT. Given the potential severity of aneurysms, if an aneurysm is suspected, imaging studies such as MRA or CTA are crucial for accurate diagnosis. In the event of nerve compression or significant hemodynamic impact, surgical intervention or endovascular treatment may be considered.

Arteriovenous etiology

- Dural arteriovenous fistulas

Dural arteriovenous fistulas (dAVFs) arising in the region of the transverse and sigmoid sinuses are a well-known cause of PT [10,34], representing up to 20% of all pulse-synchronous PT cases in some reports [3,46,47]. Arteriovenous fistulas are abnormal connections between the arterial and venous territories, causing increased venous pressure and a reversal of blood flow. Venous drainage occurs in dural venous sinuses and/or cortical/perimedullary veins [10,22,34,41,48,49], these tributary veins increase in size, dilating and causing ectasia and edema [21]. dAVFs are small tracts between meningeal arteries and small venules of the dura mater and account for approximately 10–15% of intracranial vascular malformations [50,51]. Although cerebral dAVFs frequently occur near the dural venous sinuses, they can develop anywhere in the intracranial dura mater. The most common locations include the cavernous sinus, cribriform plate, sigmoid transverse sinus, and tentorium [51,52]. Their arterial supply comes from any of the dural branches of the ICA, ECA, and VA, and in rare cases, may be fed by pial branches [51,53].

Their etiology is controversial; they may be congenital (due to aberrant connections in embryonic development), but the vast majority are acquired spontaneously, post-traumatic, or thrombotic (secondary to the recanalization of venous thrombosis) [10,22,48]. Some factors predispose to them, such as pregnancy, hypertension, arteriosclerosis, or connective tissue diseases [22].

The clinical presentation includes PT, headaches, ophthalmic symptoms (pulsatile exophthalmos, ecchymosis, vision loss), and insomnia, with more severe symptoms such as intracranial hemorrhages and non-hemorrhagic neurological deficits occurring when the fistula recruits cortical veins that extend over the brain surface. Therefore, it is crucial to rule out this etiology in cases of PT [10,22,34,41,48,49]. A thorough auscultation of the retroauricular and orbital regions should be performed, as a bruit or vascular murmur may occasionally be detected. Sometimes, digital pressure in these areas reduces the perception of the tinnitus [4,5,10,22].

**Figure 4:**
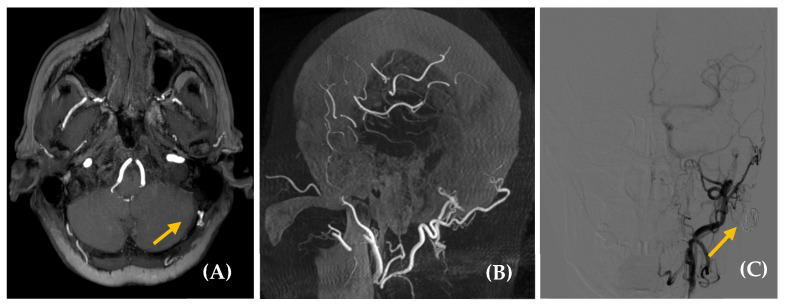
Dural arteriovenous fistula (dAVF) in a 65-year-old woman with a somatosound in the left ear, present for five years. The PT neither disappeared nor changed upon compression of the cervical vascular axis. MR angiography axial plane (image (**A**)) revealed findings suggestive of a dAVF (yellow arrow). Diagnostic–therapeutic arteriography (images (**B**), cone bean angio CT MPR in sagittal plane and (**C**), posteroanterior DSA view with injection of extracellular contrast agents) confirmed the presence of an AVF with arterial supply from the left occipital branch and superficial venous drainage to the left suboccipital region. Based on this finding, the fistula was embolized (yellow arrow, image (**C**)).

**Figure 5:**
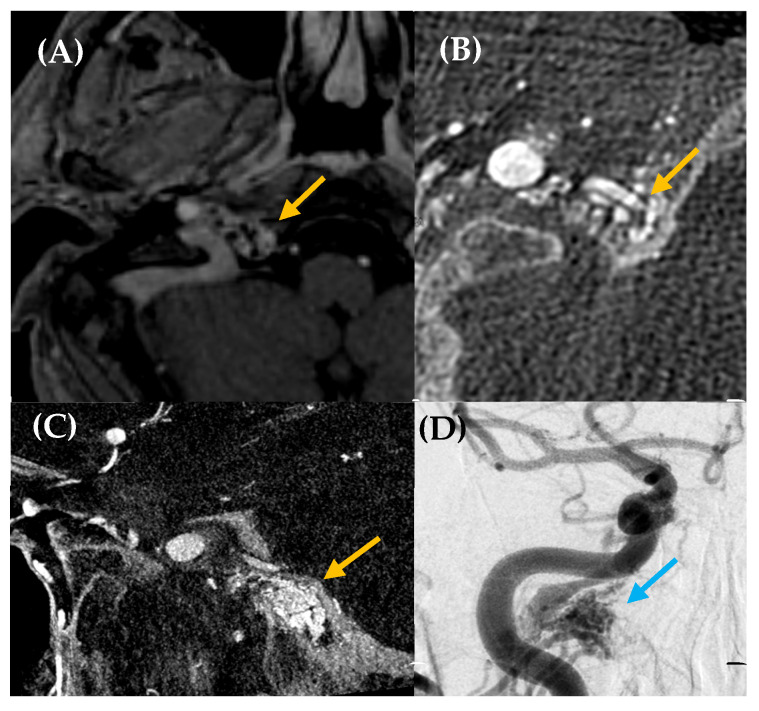
High-flow arteriovenous shunt located in the right hypoglossal canal. A 59-year-old man with a history of multiple cardiovascular risk factors and cardiac conditions presented with a somatosound in the right ear for several years. Otoscopy was unremarkable. Axial post contrast T1-weighted 3D MRI (image (**A**)) was performed, revealing a likely dural arteriovenous fistula (dAVF) in the petrous, with predominantly posteroinferior drainage (yellow arrow). Cone-beam CT angiography axial plane (images (**B**,**C**)) and DSA (image (**D**)) confirmed a high-flow arteriovenous shunt at the skull base, located in the right hypoglossal canal, measuring 15 × 16 mm (yellow arrow, images (**B**,**C**); blue arrow, image (**D**)). The fistula was primarily fed by the meningeal branch of the right occipital artery, with additional supply from neuromeningeal branches of the bilateral ascending pharyngeal arteries. Venous drainage occurred via the right inferior petrosal sinus with retrograde reflux into the cavernous sinus and the right superior ophthalmic vein, without extension into the intracranial pial veins. Embolization was performed as treatment, leading to the resolution of the patient’s PT.

Imaging studies are required for dAVF diagnosis (Figure 4 and Figure 5). MRA may accurately detect the side and presence of fistulas, making it an appropriate screening and follow-up tool [54,55]. CTA is also considered an effective diagnostic technique, with Table 6 outlining the main signs that may indicate the presence of a dAVF on this test [47,56]. In fact, some studies have suggested that 4D dynamic CTA (CTA-4D) can achieve diagnostic concordance with DSA close to 100% [57,58,59,60]. Therefore, MRA and CTA are the diagnostic tools of choice, with a high detection rate for medium- and high-flow fistulas [22]. Low-flow fistulas can often be diagnosed using the minimally invasive DSA technique, which remains the gold standard for evaluating dAVF due to its excellent spatial and temporal resolution and its ability to delineate all aspects of the complex hemodynamic features of this condition. DSA also assists in planning endovascular or surgical treatment [10,47]. Thus, our recommendation is to use MRA as a screening method for dAVF, with definitive diagnosis established via DSA, a procedure performed only to assess neurological prognosis or when therapeutic intervention, such as embolization, is anticipated [22,50].

Although many fistulas remain clinically silent and do not require treatment, the presence of cortical venous reflux, intracranial hemorrhage, and intolerable symptoms are the main indications for intervention [46,51]. Treatment typically involves an endovascular procedure or stereotactic radiosurgery to block blood flow to the fistula. In some cases, surgery may be necessary to disconnect and remove the dAVF [51].

B. Arteriovenous malformations

**Figure 6:**
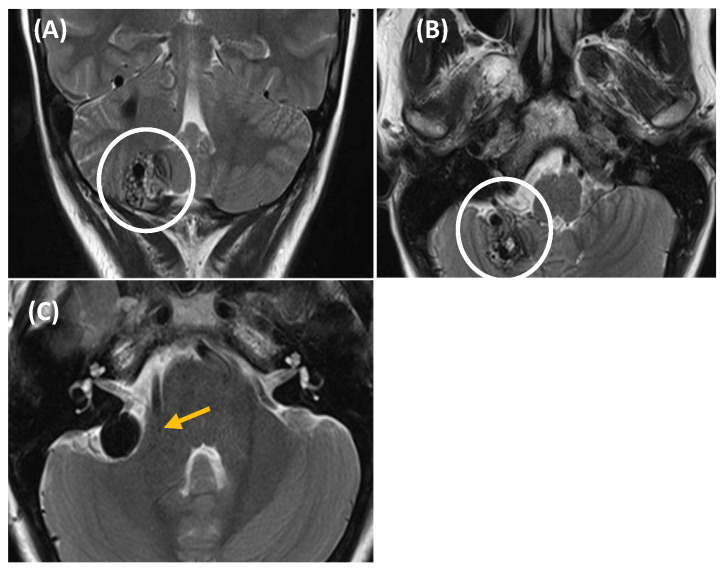
T2-weighted MRI showing a right cerebellar arteriovenous malformation (AVM) in a 44-year-old woman presented with somatosound, instability, and a sensation of pressure in the right hemiface. Coronal (image (**A**)) and axial (image (**B**,**C**)) MRI revealed a right cerebellar AVM, with a capillary nidus measuring 23 × 20 × 19 mm (outlined by a white circle in images (**A**,**B**)). The AVM showed multiple draining veins towards the anterior petrosal sinus and voluminous varicose venous dilatation (yellow arrow in image (**C**)) adjacent to the posterior border of the IAC. Given the size, location, and characteristics of the AVM, the decision was made to treat the patient using radiosurgery with Gamma Knife. This technique targets the abnormal vessels, promoting gradual occlusion of the AVM while minimizing damage to surrounding brain tissue.

Arteriovenous malformations (AVMs) consist of clusters of vessels with numerous interconnections. Similarly to dAVFs, AVMs located in the transverse and sigmoid sinuses can often cause PT [22]. They differ from dAVFs in that AVMs are lesions with an intra-axial vascular nidus, receiving arterial supply from pial arteries, without an identifiable capillary bed. Their diagnosis is typically made using MRA (Figure 6) and DSA, which are also used for prognosis assessment and treatment planning. The presence of vascular tangles on DSA is pathognomonic for AVMs, whereas associated flow or intranidal aneurysms and a pattern of diffuse leptomeningeal venous prominence, known as a pseudophlebitic pattern, are considered poor prognostic factors. The treatment is either endovascular or surgical in nature.

C. Carotid–cavernous fistulas

Carotid–cavernous fistulas (CCFs) are abnormal communications between the carotid artery and the cavernous sinus, either directly or through intradural branches of the ICA or ECA. Their etiology can be spontaneous (congenital, degenerative, or infectious), but the majority are believed to have a traumatic origin [61]. They can be classified according to Barrow et al. into direct (Type A) and indirect (Type B-C-D) types [62]. The most appropriate management for CCFs with moderate clinical symptoms and without angiographic findings indicating poor prognosis should be conservative. However, in patients for whom conservative treatment has not been effective or who show signs of poor prognosis, endovascular treatment will be performed [63,64].

Venous etiology

A variety of disorders affecting the cerebrocervical venous vasculature can also cause PT. Venous tinnitus is typically low-pitched and improves or disappears when the ipsilateral jugular vein is compressed, when the head is turned toward the healthy side, or with the Valsalva maneuver [10,65,66].

- Idiopathic intracranial hypertension

Idiopathic intracranial hypertension (IIH) is the most common cause of venous etiology in PT [22]. It is defined by the revised Friedman criteria described in Table 7 [67]. This condition is not uncommon, with an incidence of 1 case per 100,000 inhabitants per year, affecting primarily middle-aged females, obesity or rapid weight gain, black race, and menstrual irregularities [22,68,69]. The pseudotumor cerebri syndrome can be divided into primary (idiopathic intracranial hypertension) or secondary to various etiologies such as venous sinus thrombosis, medications, and medical conditions (Table 8) [70].

Clinically, it presents with headaches that worsen when lying down and visual manifestations such as diplopia due to paralysis of the VI cranial nerve, papilledema, or vision loss; and among the otoneurological symptoms, the most frequent is PT that worsens in the morning, which may be associated with sensorineural hearing loss, primarily severe and fluctuating, vertigo, and ear fullness. It has also been associated with trigeminal neuralgia or facial paralysis in some cases. Upon physical examination, PT will have the characteristics of a venous etiology, improving after jugular pressure. Fundoscopic examination is also useful, as it frequently shows papilledema [22].

IIH diagnosis will first be based on clinical suspicion following the anamnesis and physical examination; however, the diagnosis will be confirmed by an elevation of CSF pressure above 25 cm H_2_O after ruling out intracranial pathology. Therefore, an MRI study is necessary to exclude space-occupying lesions (SOLs) at the brain level. In this examination, we can often detect specific findings that suggest the diagnosis, related to the sustained increase in CSF pressure, as described in Table 9 [22,68,71]. Occasionally, when there is a high suspicion of IIH, the degree of symptoms (rated on a scale from 1 to 10 for PT) can be assessed before and after the extraction of 20 mL of CSF through lumbar puncture and obtaining venography by MRI. The closing pressure is also measured after draining the CSF, which allows us to determine whether the symptoms will improve after reducing the CSF pressure. This indicates that they will likely improve with conservative treatment. If the PT does not improve, the patient may have venous stenosis that is resistant to decreased ICP, suggesting that conservative treatment may not be effective [68,69].

The treatment will initially be conservative, involving hygienic-dietary recommendations such as weight reduction, which will be sufficient to reverse the symptoms in many patients. If there is no improvement, pharmacological measures will be employed, such as diuretics (acetazolamide or furosemide), which help decrease CSF production and alleviate hyperpressure. If there is still no improvement, a peritoneal shunt may be placed, or even an optic nerve sheath fenestration could be required in cases of progressive visual loss risk [22]. In selected patients with transverse sinus stenosis and demonstrated pressure gradients, endovascular treatment with angioplasty and stenting may be useful [72].

B. High-riding/dehiscent jugular bulb

**Figure 7:**
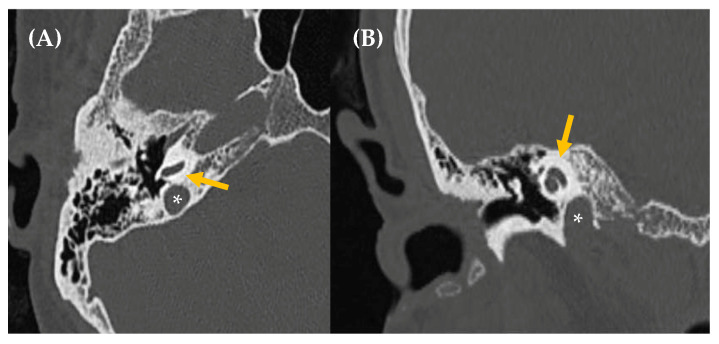
CT imaging (image (**A**), axial section/image (**B**), coronal section) reveals asymmetry in size between both jugular bulbs, more prominent on the right side, which reaches a slightly higher position than the contralateral one, up to the height of the basal turn of the cochlea, without reaching the height of the IAC. The jugular wall is continuous, with no evidence of dehiscence (asterisk: jugular bulb/yellow arrow: cochlea).

Some venous-origin PT cases may be related to a dehiscence or loss of the bony covering between a venous sinus and the mastoid air cells. In these instances, the sound of normal venous flow is excessively transmitted to the cochlea via the mastoid air cells. These dehiscences are a cause of retrotympanic vascular mass and can be identified through high-resolution temporal bone CT (Figure 7). One such case occurs when dehiscence happens at the level of the sigmoid (jugular) plate, which normally separates the jugular bulb from the hypotympanum and middle ear cavity. In other cases, the jugular bulb may be prolapsed, meaning it is located higher than usual, possibly accompanied by a dehiscence in the sigmoid plate or not. The transverse level above which a jugular bulb is considered prolapsed or high has been variably defined: when the jugular bulb apex is at the level of the floor of the IAC or 2 mm below it, if it exceeds the superior tympanic annulus, if it reaches the basal turn of the cochlea or the inferior edge of the round window niche, if it is above the floor of the external auditory canal (EAC) or inferior edge of the tympanic annulus, or if it is above the cochlear aqueduct [73]. Depending on the definition used, prevalence varies across studies, ranging from 6% to 34%. Jugular bulb ectasias or dehiscences may be treated using endovascular or surgical approaches, with careful consideration of the risk–benefit balance [71].

C. Other

Other potential venous-origin etiologies of PT include aneurysms in venous sinuses near the inner ear, such as a lateral sinus aneurysm, which can cause a prolapse of the sinus wall into the mastoid air cells and is treated endovascularly. Additionally, dehiscence or dilation of a mastoid or posterior condylar emissary vein (causing turbulence due to bidirectional flow from the absence of valves) may also lead to PT. Treatment options for these cases include selective occlusion of the emissary vein using coils in endovascular procedures, surgical intervention to cover the bony defect (if associated with dehiscence), or observation with lifestyle modifications [71].

##### Non-Vascular Etiology

Neoplastic origin

Neoplasms or vascular masses at the skull base have been described as potential causes of PT. Table 2 describes the main tumors that can cause PT.

- Paraganglioma

Paraganglioma is the most common vascular tumor at the skull base causing PT [3,5,10,34,37,41], and it occurs more frequently in women between the fifth and sixth decades of life. This slow-growing neuroendocrine neoplasm originates from glomus chromaffin cells derived from the embryonic neural crest and can be associated with pathogenic variants in genes encoding the succinate dehydrogenase enzyme complex. While the majority, up to 97%, are benign, they can still be locally destructive, leading to erosion of adjacent bony structures [3,37]. Paragangliomas are highly vascular tumors that, in the head and neck region, develop along the cervical vascular axes. The most frequent locations include the middle ear (along the cochlear promontory, as they arise from the tympanic nerve or Jacobson’s nerve, a branch of the IX cranial nerve), the jugular foramen, vagal nerve, and the carotid body (known as tympanic, jugular, vagal, and carotid body tumors, respectively). Tympanic, jugular, or jugulotympanic paragangliomas can cause PT due to their proximity to the cochlea; however, carotid body or vagal paragangliomas typically do not cause PT [3,10,37].

**Figure 8:**
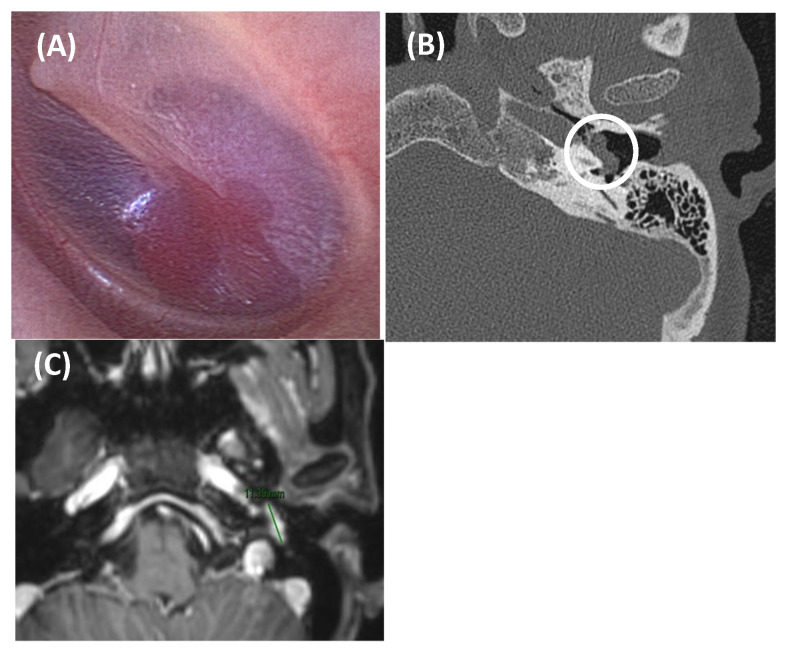
Tympanic paraganglioma in an 81-year-old woman presented with somatosound in the left ear, displaying arterial characteristics. Otoscopy revealed a red retrotympanic mass (image (**A**)). An axial section of a CT scan (Image (**B**)) showed a small soft tissue tumor in the left ear over the cochlear promontory (outlined by a white circle), about 8.3 mm in anteroposterior diameter, suggestive of tympanic paraganglioma. Axial post gadolinium T1-weighted MRI scan (Image (**C**)) confirmed a mass at this level with intense gadolinium enhancement (green line). In this case, due to the patient’s age and associated risks, a decision was made to monitor the condition with regular follow-up.

**Figure 9:**
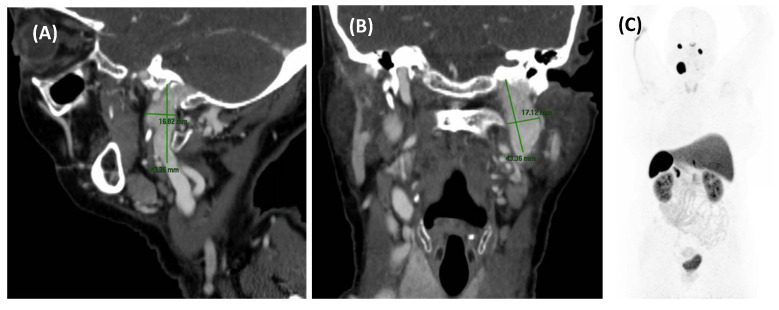
A CT scan (sagittal section, image (**A**); coronal section, image (**B**)) in a 76-year-old woman with somatosound in the left ear for 3 years. It reveals an area of intense contrast uptake, with a larger craniocaudal diameter, located at the left basal cranial level which, due to its location, is suggestive of a jugular paraganglioma. The SPECT-CT scan with 111In-pentetreotide (image (**C**)) did not show other foci of tracer uptake, suggesting the absence of a neuroendocrine tumor with positive somatostatin receptor expression elsewhere in the body. This patient was treated with radiotherapy with a good response.

**Figure 10:**
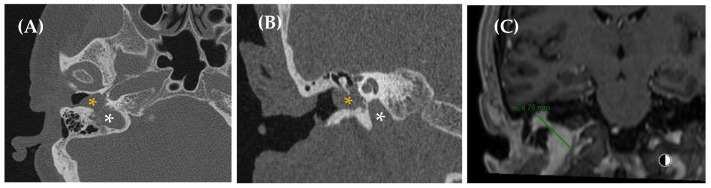
A jugulotympanic paraganglioma in a 60-year-old woman presented with somatosound in the right ear, which increased with exercise and had been present for a long time. The CT scan (axial section, image (**A**); coronal section, image (**B**)) revealed a tumor at the level of the jugular bulb (white asterisk) with a tympanic component (orange asterisk). T1-weighted coronal post gadolinium MRI (image (**C**)) showed a hypercapillary lesion in the jugular bulb on the right side, extending into the tympanic cavity with avid enhancement (green line). The component of the lesion located in the jugular bulb measures around 18 × 12 mm and, in the tympanic cavity, 10 × 14 mm, suggesting a diagnosis of right jugulotympanic paraganglioma.

Usually, the initial clinical presentation of a paraganglioma is PT, which worsens with physical activity and does not change with compression of the jugular vein or occipital artery. A pulsatile red mass may be visible behind the tympanic membrane during otoscopy in patients with tumors affecting the tympanic cavity. Imaging studies should include a CT scan, which typically reveals a rounded soft tissue mass in the middle ear along the cochlear promontory, characteristic of tympanic paragangliomas. In larger paragangliomas (jugular or jugulotympanic), CT often shows bone erosion of adjacent structures. MRI with gadolinium contrast typically demonstrates an intensely enhancing mass, with larger tumors showing a distinctive heterogeneous “salt and pepper” appearance on T2 images (Figure 8, Figure 9 and Figure 10) [3,4,5,10,37].

Treatment for strictly tympanic paragangliomas is surgical, often requiring preoperative angiography with embolization to prevent significant bleeding. In some cases, radiotherapy effectively controls tumor growth, particularly in cervical paragangliomas. Depending on the stage of the disease, treatment may involve resection, embolization, external radiotherapy, or systemic radionuclide therapy [3,10,12,37].

Temporal bone abnormalities

- Superior canal dehiscence syndrome

SCDS refers to the presence of a bone defect in the bony covering of the superior semicircular canal (SSC). Normally, for hearing to occur, the inner ear operates via a “two-window mechanism,” where sound enters through the oval window, is converted into neural activity, and exits through the round window. However, when SCDS is present, a “third window” allows sound energy to travel between the SSC and the middle cranial fossa. This defect can be either congenital or acquired, typically post-traumatic. Clinically, patients may present with conductive hearing loss, ear fullness, autophony (due to hypersensitivity to bone-conducted sounds), and vertigo triggered by loud sounds or pressure changes (e.g., Valsalva maneuver). In some cases, patients may report PT, which could be attributed to sound transmission from the dural vessels of the middle cranial fossa or the superior petrosal sinus [33,74]. Additionally, there is a suggested potential relationship between SCDS and IIH, which may also contribute to the development of PT. CT scans reconstructed using Pöschl and Stenvers views reveal the dehiscence. However, radiological findings are not considered a unique diagnostic criterion for SCDS; rather, other tests such as vestibular-evoked myogenic potentials (VEMPs) may be required to fulfill the diagnostic criteria (Table 10) [75]. Treatment options include conservative management or surgical repair [33].

B. Other

Otosclerosis and bone dysplasias affecting the temporal bone, such as Paget’s disease or osteopetrosis, have also been associated with the development of PT, likely due to increased bone vascularization and the formation of arteriovenous fistulas within the temporal bone [1,10]. Otosclerosis is a bone disorder that leads to progressive bone remodeling and resorption with vascular proliferation in the temporal bone. It causes the replacement of normally dense osseous labyrinth with vascular haversian bone, which can appear radiolucent on CT scans during the active phase of the disease [1,4,5,10,22,37]. Clinically, it results in conductive or mixed hearing loss and is often associated with tinnitus, which can sometimes be pulsatile, especially in the early stages of the disease. In some cases, stapedectomy may resolve the symptoms.

Similarly, Paget’s disease can also cause conductive or mixed hearing loss, with up to 10% of affected patients experiencing PT [22]. Paget’s disease is a non-tumorous disorder of bone remodeling that leads to disorganized and hypervascular bone formation. On CT scans, “Pagetoid bone” appears with a characteristic cotton–wool texture [1,3,5,10,22,34,37].

Rhythmic tinnitus

Tinnitus may present as continuous or rhythmic tones similar to PT but with a different frequency from the heartbeat, often associated with myoclonic mechanisms. These types of tinnitus are sometimes referred to as pseudo-pulsatile because they could be confused with PT. These are caused by involuntary muscle contractions, particularly in the middle ear or pharynx. The most common forms include soft palatal tremors (palatal myoclonus), involving the muscles of the soft palate, and myoclonus of the middle ear muscles (tensor tympani and stapedius). This uncontrolled muscle contraction generates non-pulsatile sounds, often described as bursts or rapid, rhythmic noises. Clinically, the patient will describe the sound as brief and resembling the “clicking of a typewriter” [22,32]. However, it is important to rule out central causes such as multiple sclerosis, brainstem infarction, or cerebellar disorders as possible etiologies. Diagnosis may sometimes reveal fasciculations at the tympanic membrane during otoscopy (middle ear muscle myoclonus), or movements of the soft palate muscles via fibroendoscopy, in which asking the patient to open their mouth can reduce the tinnitus (soft palate muscles myoclonus), or movements in the soft palate may be observed during an oral cavity exam (palatal myoclonus) [22]. Additional diagnostic steps might include auscultation, tympanometry, and blood tests for calcium, potassium, and magnesium levels, as abnormalities in these can be related to the condition. Pharmacological treatments, such as benzodiazepines or carbamazepine, have been reported, as well as surgical options like tenotomy. However, the most common treatment currently involves therapeutic advice, explanation of the pathology, and prognosis and reassurance; and if it is very limiting, repeated botulinum toxin injections, depending on the progression of the condition. Trans-electrical nerve stimulation (TENS) has also been described as a potentially effective method for treating myoclonus [22].

Miscellanea

Other potential origins of somatosounds include any conditions that cause capillary hyperemia in the petrous temporal bone (such as microfistulas of the inner ear), somatosounds from bone clicks (typical of temporomandibular joint disorders), respiratory mechanisms (such as patulous eustachian tube syndrome), or any disorders that result in conductive hearing loss (such as chronic otitis media).

#### 3.2.2. Bilateral Pulsatile Tinnitus

When the patient presents bilateral PT, the investigation should be oriented towards ruling out systemic pathology rather than vascular issues. In cases of bilateral PT, we must consider conditions that cause an increase in cardiac output, such as anemia, thrombocythemia, hyperthyroidism, pregnancy, and valvular heart diseases. If all of these are normal, and symptoms worsen, or neurological signs appear (severe headache, vertigo, visual changes) we can proceed with an imaging protocol in order to rule out other potential causes.

## 4. Discussion

In general, with a thorough investigation, an underlying etiology of PT can be identified in over 70–80% of cases [24,41]. As shown in Table 9 [25], the imaging study most effective in detecting the underlying pathology causing PT is an MRI combined with MRA, followed by CT or CTA. Indeed, the pathologies that should be prioritized for exclusion due to their associated morbidity and mortality, such as dAVFs or AVMs, can be initially evaluated with these imaging techniques. Therefore, the recommended diagnostic protocol is to initially perform an MRI + MRA, arterial or venous depending on diagnostic suspicion. If these tests yield normal results, a CT scan should then be considered to assess other potential etiologies, such as aberrant ICA, high-riding, or dehiscent jugular bulb, or pathologies involving the temporal bone or ear cavity. Additionally, as noted in Table 11, Doppler ultrasound is not the preferred method for ruling out vascular stenosis. Instead, in cases with a high clinical suspicion of vascular involvement, an MRA (arterial or venous depending on the etiological suspicion of the origin) should be prioritized in the diagnostic protocol for PT. DSA remains the gold standard for detecting AVFs and AVMs due to its superior spatial and temporal resolution. Therefore, when other imaging studies raise suspicion, this examination should be requested to evaluate poor prognostic factors and to plan appropriate treatment.

If we review the relevant literature [3,22,26,41,46,69,76,77], the most common cause of PT is arterial vascular stenosis. With regard to tinnitus of venous etiology, IIH is the most frequent cause (corresponding to the second most frequent cause of PT), along with other venous malformations and venous anatomical variants. Another relatively frequent etiology that must be ruled out due to its potential severity is AVF (dural or direct), which accounts for more than 9% of PT cases (Table 12) [3,22,26,41,46,69,76,77]. However, there is considerable variability among studies regarding these findings. What is widely accepted is that IIH and vascular stenosis are the primary causes of PT. This underscores the importance of guiding the examination to differentiate between arterial and venous etiologies and, based on these findings, requesting appropriate tests with a clinical judgment focused on the suspected pathology.

**Figure 11:**
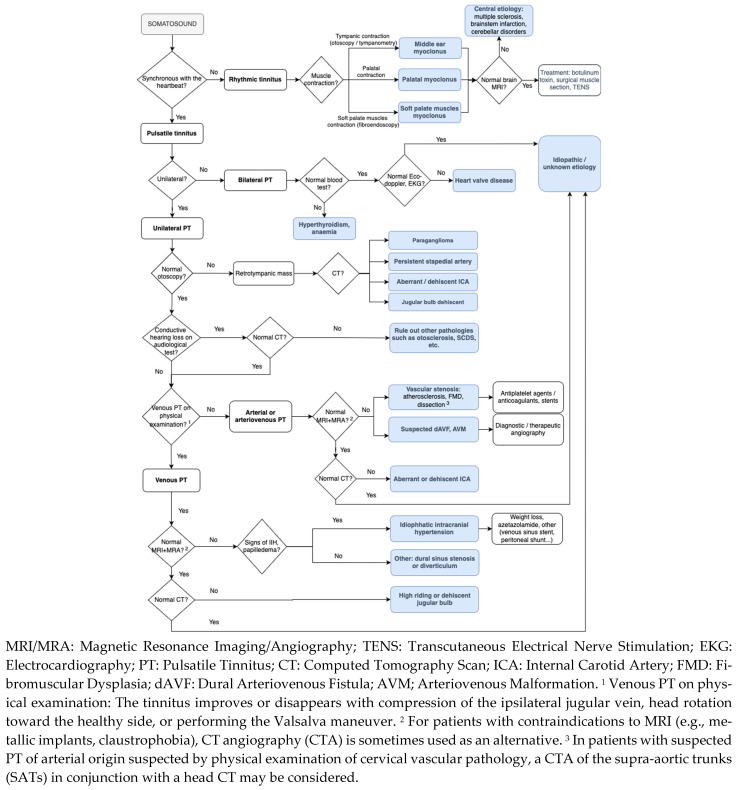
Clinical approach of pulsatile tinnitus.

A diagnostic algorithm for PT is proposed in Figure 11 [22,34,65] and a summary of the diagnostic protocol established in our center is shown in Table 13.

One of the main limitations of the manuscript is that including studies from the past 30 years may introduce considerable heterogeneity, due to significant changes in clinical management and diagnostic technologies for PT. Studies conducted earlier within the 30-year time frame may not reflect current clinical practices for managing PT. It is important to take into account the potential impact of this time cut-off on the generalizability of the study’s findings. Since this is a scoping review, which does not assess the quality of evidence or provide a quantitative synthesis, the conclusions may be diffuse or open to interpretation. To date, a limited number of prospective studies have addressed this topic. There is an insufficient amount of quantitative data to support the development of a meta-analysis, which would provide the highest level of evidence for formulating robust recommendations regarding the diagnostic management of PT. However, given the current limitations in the available research, further prospective studies with direct comparisons are needed in order to establish standardized diagnostic protocols. Ideally, these efforts should culminate in clinical guidelines developed by international organizations focused on the clinical management of PT, providing a unified framework for clinicians dealing with this symptom.

## 5. Conclusions

Somatosounds are sounds perceived by the ear that originate within the body and are capable of stimulating cochlear mechanics. They require careful evaluation, as an inaccurate or missed diagnosis can have serious consequences given that their underlying causes may involve significant morbidity and mortality, such as arteriovenous fistulas. In most cases, a potentially treatable cause can be identified through a detailed medical history, thorough cervical and cranial auscultation, and comprehensive radiological assessment. This evaluation should be focused based on whether the tinnitus is arterial or venous in nature, if an audible bruit is present, or if a retrotympanic mass is detected. Treating the underlying cause often results in the resolution of pulsatile tinnitus.

## Figures and Tables

**Table 1:** Main causes of retrotympanic vascular abnormalities.

Aberrant and/or dehiscent internal carotid artery Persistent stapedial artery Tympanic/jugular paraganglioma Jugular bulbar abnormalities (high-riding, dehiscence, or diverticulum)

**Table 2:** Causes of pulsatile tinnitus by etiology groups.

Etiology	
Vascular	
Arterial	Vascular stenosis or occlusion: Atherosclerosis Dissection Fibromuscular dysplasia Variants of normal skull base anatomy: Aberrant or dehiscent internal carotid artery Persistent stapedial artery Arterial compression of vestibulocochlear nerve Cerebral aneurysms Dolichoectasia
Arteriovenous	Dural arteriovenous fistula Arteriovenous malformation Carotid–cavernous fistula
Venous	Idiopathic intracranial hypertension Transverse or sigmoid sinus stenosis Dural venous diverticulum Abnormal jugular bulb, such as high-riding bulb, diverticulum or dehiscence Prominent/dilated emissary veins (e.g., mastoid, condylar)
Non-Vascular	
Structural	
Neoplasm	Paraganglioma Vestibular schwannoma [33] Skull base meningioma Endolymphatic sac neoplasm (frequently associated with Von Hippel–Lindau disease) Skull base metastasis Other: cavernous middle ear hemangioma, hemangiopericitoma, facial nerve hemangioma, etc.
Temporal bone abnormalities	Superior semicircular canal dehiscence Otosclerosis Paget’s disease Sigmoid sinus dehiscence/dehiscent jugular bulb Petrous carotid canal dehiscence
Other	
High cardiac output	Hyperthyroidism Anemia Pregnancy Valvular heart disease (aortic stenosis)
Miscellanea	Microfistula of the inner ear Chronic otitis media

**Table 3:** Radiological signs suggestive of a persistent stapedial artery.

Enlarged facial nerve canal or a separate canal running parallel to the facial nerve. Aplastic or hypoplastic spinous foramen. Middle meningeal artery arising from the ophthalmic artery or absence of the proximal branches of the middle meningeal artery.

**Table 4:** Chavda and McDermott classification of AICA loops [39].

Type I: lying only in the CPA, but not entering the IAC Type II: entering but not extending > 50% of the length of the IAC Type III: entering and extending > 50% of the length of the IAC

CPA: cerebellopontine angle; IAC: internal auditory canal.

**Table 5:** Møller’s ABR criteria for diagnosing a microvascular conflict of the eighth nerve [43,44].

Ipsilateral IPL I-III ≥ 2.3 ms Contralateral IPL III-V ≥ 2.2 ms IPL I-III difference ≥ 0.2 ms IPL III-V difference ≥ 0.2 ms IPL I-III difference ≥ 0.16 ms if low or absent peak II IPL III-V difference ≥ 0.16 ms if low or absent peak II Peak II amplitude < 33%

IPL: interpeak latency; ms: millisecond.

**Table 6:** Signs that may indicate the presence of a dAVF in CTA [47,56].

Sensitive and specific marker Abnormally enlarged arterial feeding vessels Less sensitive markers Asymmetrical increased draining veins The presence of transcalvarial channels “Shaggy” appearance of the tentorium and adjacent venous sinuses The presence of prominent cortical venous channels

**Table 7:** Suggested update to revised Friedman criteria for diagnosis of idiopathic intracranial hypertension [67].

Presence of at least 2 of 3 signs of elevated intracranial pressure ^1^ (1) Papilledema ^2^ (2) Elevated lumbar puncture opening pressure ≥ 25 cm CSF in adults measured before withdrawal of CSF in a relaxed patient placed in left, lateral decubitus position with neck and legs extended. (3) Presence of ≥ 3 neuroimaging signs (cerebral MRI and MRI- or CT venography described by a neuroradiologist) ^3^ Partial empty sella (≥ grade 3) Uni- or bilateral flattening of the posterior aspect of the globe Uni- or bilateral distension of the perioptic subarachnoid space Bilateral sinus venous stenoses (ITSS score ≥ 4) Normal neurological examination except for cranial nerve abnormalities. Cerebral MRI and venography (MRI/CT) without hydrocephalus, structural lesions or venous sinus thrombosis. Normal CSF composition. No secondary cause of elevated ICP. Secondary ICP elevation, satisfying A–D, is referred to as secondary pseudotumor cerebri syndrome ^4^.

^1^ IIH is probable in case of stand-alone papilledema if B–E are met. Consider new measurement of OP/ICP-monitoring. ^2^ Pseudo-papilledema should be excluded by a neuro-ophthalmologist. Papilledema can be asymmetrical/unilateral. ^3^ Thin-cut orbital MRI-sequences are highly recommended. Specificity is only sufficiently high if ≥ 3 neuroimaging signs are present. Neuroimaging signs are defined according to the following: a. Minimum requirement for pituitary pathology is suprasellar herniation of CSF > 1/3 into the sella turcica with or without compression of pituitary gland tissue; b. Distension of the perioptic subarachnoid space: The space is > 2 mm in the coronal/axial plane; c. Flattening of the posterior aspect of the globe is a qualitative evaluation (neuroradiologist); d. Bilateral sinus venous stenoses: ITSS score ≥ 4. Stenosis was graded 1–4 on each side: 1 ≤ 33% stenosis, 2 = 33–66% stenosis, 3 ≥ 66% stenosis and 4 = hypoplasia or agenesia. Hypoplasia was defined as the full-length diameter being < 1/3 of the superior sagittal sinus. The ITSS score is calculated by multiplying the two grades. ^4^ Secondary intracranial hypertension, pseudotumor cerebri syndrome, can be indistinguishable from IIH. IIH is rare in post-menopausal women, patients with BMI < 25 and in men. Secondary ICP-elevation should be ruled out by a specialist. CSF: cerebrospinal fluid; CT: computed tomography; ICP: intracranial pressure; ITSS: index of transverse sinus stenosis; MRI: magnetic resonance imaging; OP: opening pressure.

**Table 8:** Classification of pseudotumor cerebri syndrome.

Primary Pseudotumor Cerebri (Idiopathic Intracranial hypertension)
Include patients with obesity, recent weight gain, polycystic ovary syndrome, and thin children.
Secondary pseudotumor cerebri
Cerebral venous abnormalities Cerebral venous sinus thrombosis Bilateral jugular vein thrombosis or surgical ligation Middle ear or mastoid infection Increased right heart pressure Superior vena cava syndrome Arteriovenous fistulas Decreased CSF absorption due to previous intracranial infection or subarachnoid hemorrhage Hypercoagulable state Medications and exposures Antibiotics: Tetracycline, minocycline, doxycycline, nalidixic acid, sulfa drugs Vitamin A and retinoids: Hypervitaminosis A, isotretinoin, all-trans retinoic acid for promyelocytic leukemia, excessive liver ingestion Hormones: human growth hormone, thyroxine (in children), leuprorelin acetate, levonorgestrel (Norplant system), anabolic steroids Withdrawal from chronic corticosteroids Lithium Chlordecone Medical conditions Endocrine disorders: Addison’s disease, hypoparathyroidism, hypothyroidism, systemic lupus erythematosus Hypercapnia: sleep apnea, Pickwickian syndrome Other: anemia, renal failure, Turner syndrome, Down syndrome, etc.

**Table 9:** Radiological signs on MRI suggesting idiopathic intracranial hypertension.

Empty sella Decreased ventricular size, increased subarachnoid space Uni- or bilateral transverse sinus stenosis (prevalence up to 90% in idiopathic intracranial hypertension): the most frequent location is in the distal region of the transverse sinuses or at their junction with the sigmoid sinuses Distension of the perioptic space and contrast enhancement of the optic nerves Enlargement of Meckel’s cave Skull base meningoceles

**Table 10:** Diagnostic criteria for superior semicircular canal dehiscence syndrome [75].

The diagnosis of superior semicircular canal dehiscence syndrome requires all of the following criteria: At least 1 of the following symptoms consistent with the presence of a “third mobile window” in the inner ear: Bone conduction hyperacusis Sound-induced vertigo and/or oscillopsia time-locked to the stimulus Pressure-induced vertigo and/or oscillopsia time-locked to the stimulus Pulsatile tinnitus At least 1 of the following signs or diagnostic tests indicating a “third mobile window” in the inner ear: Nystagmus characteristic of excitation or inhibition of the affected superior semicircular canal evoked by sound, or by changes in middle ear pressure or intracranial pressure Low-frequency negative bone conduction thresholds on pure tone audiometry Enhanced VEMP responses (low cervical VEMP thresholds or high ocular VEMP amplitudes) High resolution temporal bone CT imaging with multiplanar reconstruction demonstrating dehiscence of the superior semicircular canal Not better accounted for by another vestibular disease or disorder

**Table 11:** The value of different imaging modalities for the detection and characterization of the different pathologies that can cause PT (modified from Pegge et al. [25]).

Pathology	CT	CTA	4D-CTA	MRI	MRA	DSA	Duplex Ultrasound
Tympanic cavity pathology	+++	+++	+++	-	-	-	-
Temporal bone pathology (otosclerosis, Paget disease)	+++	+++	+++	+	-	-	-
Paraganglioma	++	++	++	+++	+++	+	+
Hypervascular skull base tumors	+	++	+	+++	±	-	-
Vascular channel dehiscence or variant	+++	+++	+++	-	-	-	-
Aberrant ICA or persistent stapedial artery	+++	+++	+++	-	±	+++	-
Vascular loops, neurovascular conflict	-	+	+	+++	++	-	-
Arteriovenous fistula	-	+	+++	±	++	+++	+
Arteriovenous malformation	-	++	++	++	++	+++	+ (superficial)
Vascular stenosis (e.g., atherosclerosis, FMD or dissection)	-	++	+	±	+++	+	++
Idiopathic intracranial hypertension	-	-	-	+++	±	-	-

+++: most optimal; ++: good; +: moderate; ±: indirect signs; -: not suitable; ICA: internal carotid artery; FMD: fibromuscular dysplasia.

**Table 12:** Total of patients (*n*) and relative frequency (%) of main causes of pulsatile tinnitus.

Location	Etiology	Dietz 1994 [76]	Sismanis 1998 [69]	Waldvogel 1998 [46]	Sonmez 2007 [3]	Herraiz 2007 [22]	Mattox 2008 [77]	Hofmann 2013 [41]	Bae 2015 [26]	Total
Arterial		4 (8.2%)	33 (22.8%)	19 (22.6%)	20 (27.1%)	22 (27.6%)	14 (26%)	11 (14.3%)	8 (14.1%)	131 (21.1%)
	Arterial stenosis	2 (4.1%)	24 (16.6%)	17 (20.2%)	16 (21.6%)	15 (18.8%)	13 (24.1%)	7 (9.1%)	4 (7%)	98 (15.8%)
	Aneurysms (ICA, VA)	0 (0%)	2 (1.4%)	1 (1.2%)	3 (4.1%)	1 (1.3%)	0 (0%)	3 (3.9%)	3 (5.3%)	13 (2.1%)
	Anatomical variants (aberrant ICA, PSA, carotid–cochlear dehiscence)	2 (4.1%)	7 (4.8%)	1 (1.2%)	1 (1.4%)	6 (7.5%)	1 (1.9%)	1 (1.3%)	1 (1.8%)	20 (3.2%)
Arteriovenous		19 (39.7%)	25 (17.3%)	28 (33.3%)	4 (5.4%)	16 (20.1%)	0 (0%)	27 (35.1%)	7 (12.3%)	126 (20.3%)
	dAVF	10 (20.4%)	3 (2.1%)	17 (20.2%)	2 (2.7%)	3 (3.8%)	0 (0%)	6 (7.8%)	5 (8.8%)	46 (7.4%)
	Direct AVF	3 (6.1%)	0 (0%)	6 (7.1%)	0 (0%)	0 (0%)	0 (0%)	2 (2.6%)	0 (0%)	11 (1.8%)
	AVM	1 (2%)	1 (0.7%)	0 (0%)	0 (0%)	0 (0%)	0 (0%)	1 (1.3%)	0 (0%)	3 (0.4%)
	Vascularized neoplasms (paraganglioma, skull base tumors)	5 (10.2%)	17 (11.7%)	5 (6%)	2 (2.7%)	2 (2.5%)	0 (0%)	12 (15.6%)	2 (3.5%)	45 (7.3%)
	Capillary hyperemia (acute otitis media, otosclerosis)	0 (0%)	4 (2.8%)	0 (0%)	0 (0%)	11 (13.8%)	0 (0%)	6 (7.8%)	0 (0%)	21 (3.4%)
Venous		5 (10.2%)	61 (42.1%)	7 (8.3%)	25 (33.8%)	11 (13.8%)	24 (44.5%)	17 (22.1%)	28 (49.2%)	1578 (28.7%)
	IIH	0 (0%)	61 (42.1%)	6 (7.1%)	0 (0%)	8 (10%)	1 (1.9%)	6 (7.8%)	1 (1.8%)	83 (13.4%)
	Anatomical variants (high-riding jugular bulb, jugular bulb dehiscence, transverse or sigmoid sinus diverticulum)	5 (10.2%)	0 (0%)	1 (1.2%)	25 (33.8%)	3 (3.8%)	23 (42.6%)	11 (14.3%)	27 (47.4%)	95 (15.3%)
Other		0 (0%)	13 (9%)	3 (3.6%)	1 (1.4%)	21 (26.3%)	1 (1.9%)	7 (9.1%)	4 (7%)	175 (8.1%)
	SCDS	0 (0%)	0 (0%)	0 (0%)	0 (0%)	0 (0%)	1 (1.9%)	4 (5.2%)	0 (0%)	5 (0.8%)
	Other causes	0 (0%)	13 (9%)	3 (3.6%)	1 (1.4%)	21 (26.3%)	0 (0%)	3 (3.9%)	4 (7%)	45 (7.3%)
Idiopathic		21 (42.9%)	13 (9%)	27 (32.1%)	24 (32.4%)	10 (12.5%)	15 (27.8%)	15 (19.5%)	10 (17.5%)	135 (21.8%)
Total patients (*n*)		49	145	84	74	80	54	77	57	620

ICA: internal carotid artery; VA: vertebral artery; PSA: persistent stapedial artery; dAVF: dural arteriovenous fistula; AVF: arteriovenous fistula; AVM: arteriovenous malformation; IIH: idiopathic intracranial hypertension; SCDS: superior canal dehiscence syndrome.

**Table 13:** Summary of diagnostic imaging protocol at Hospital Universitario Virgen de las Nieves, Granada (Spain).

Non-Pulsatile Tinnitus
Unilateral (with or without unilateral or asymmetrical sensorineural hearing loss)
Request MRI with and without contrast of the skull and IAC/CPA
The aim of diagnostic imaging is to rule out IAC/CPA pathology, most commonly a VS. 6% of VS present with unilateral tinnitus, and this percentage is significantly higher if associated with ipsilateral sensorineural hearing loss [20]. MRI of the IAC should be requested for all patients with unilateral non-pulsatile tinnitus, even if there is no hearing loss. All cases of sudden hearing loss require an MRI, as 20% of patients with vs. may present with sudden hearing loss, although only 4% of sudden hearing loss cases are caused by a vs. [17,18,19,20]. Asymmetrical hearing loss is defined as a difference of 20 or more decibels (dB) in two consecutive frequencies (500, 1000, 2000, and 4000 Hz), with normal hearing in the contralateral ear, or a difference of ≥ 15 dB in two or more frequencies, or a difference greater than 15% in speech discrimination between the two ears [14,15,16].
Bilateral
In patients with bilateral tinnitus and normal hearing or symmetrical hearing loss, imaging tests are generally not required, as it is now well understood that imaging results are usually normal.
Pulsatile Tinnitus
Unilateral
Request cranial MRI with IAC/CPA sequences and MRA of the cranial vessels and the circle of Willis
Tinnitus of venous origin is typically low-pitched and improves or disappears with ipsilateral jugular vein compression, head rotation toward the healthy side, or during a Valsalva maneuver. In rare cases where compression of the vascular axis worsens the tinnitus, a condylar venous overflow or carotid stenosis should be considered. When assessing, note whether the clinical suspicion leans toward arterial or venous tinnitus. If MRI is normal, or in patients with conductive hearing loss and normal otoscopy, or when a retrotympanic mass is visible on otoscopic examination, a high-resolution CT of the head and temporal bone should be considered. For patients with contraindications to MRI (e.g., metallic implants, claustrophobia), CTA is sometimes used as an alternative. If cervical vascular pathology is suspected, a CTA of the supra-aortic trunks (SATs) in conjunction with a head CT may be considered. DSA is now generally reserved for situations where there is strong suspicion of an arterial cause or high likelihood of dAVF, and it can also be used as a therapeutic tool.
Bilateral
Anemia, thrombocythemia, hyperthyroidism, pregnancy, and valvular heart disease must be ruled out. Laboratory tests, including complete blood count and thyroid hormones levels, should be evaluated. If normal, an echocardiogram should be performed to rule out aortic stenosis. If all of these are normal, and symptoms worsen, or neurological signs appear (severe headache, vertigo, visual changes), we can proceed with an imaging protocol in order to rule out other potential causes.

MRI: Magnetic Resonance Imaging; IAC: Internal Auditory Canal; CPA: Cerebellopontine Angle; VS: Vestibular Schwannoma; MRA: Magnetic Resonance Angiography; CT: Computed Tomography Scan; CTA: Computed Tomography Angiography; DSA: Digital Subtraction Angiography; dAVF: dural Arteriovenous Fistula.

## Data Availability

No new data were created or analyzed in this study.

## Supplementary Materials

The following supporting information can be downloaded at: https://www.mdpi.com/article/10.3390/jcm14134428/s1, Table S1: Outcome data extracted from each included source of evidence. This table was prepared in accordance with PRISMA-ScR item 17.
