# Supplementary material for: Pulsatile Tinnitus: A Comprehensive Clinical Approach to Diagnosis and Management
Source: J Clin Med. 2025 Jun 22;14(13):4428. doi: 10.3390/jcm14134428 (PMC12250305; doi:10.3390/jcm14134428)
Supplement: Supplementary file 1 [file jcm-14-04428-s001.zip › jcm-3617843-supplementary.pdf]

Supplementary Table S1. Outcome data extracted from each included source of evidence. This table was prepared in accordance with PRISMA-ScR item 17.

Author(s)	Year	Title of the article	Type of article	Target population	Relevant outcomes data extracted
**Weissman et al. [4]**	2000	Imaging of tinnitus: a review.	Review article	Patients with pulsatile or nonpulsatile tinnitus	Basic concepts of tinnitus in general, pulsatile and non-pulsatile tinnitus.
**Lockwood et al. [8]**	2002	Tinnitus	Review article	Patients with pulsatile or nonpulsatile tinnitus	Concepts of tinnitus, particularly epidemiology and definition.
**Bauer et al. [6]**	2004	Mechanisms of tinnitus generation	Narrative review	Individuals with tinnitus	Concepts of tinnitus, particularly epidemiology and definition.
**Branstetter et al. [1]**	2006	The radiologic evaluation of tinnitus	Review article	Patients with tinnitus undergoing imaging	Concepts of tinnitus: pulsatile or not. Radiological imaging in treatable causes of pulsatile tinnitus.
**Liyanage et al. [2]**	2006	Pulsatile tinnitus	Narrative review	Patients with PT	Basic concepts of tinnitus in general.
**Krishnan et al. [7]**	2006	CT arteriography and venography in pulsatile tinnitus: preliminary results.	Original research (prospective study)	Patients with PT	Concepts of tinnitus, particularly epidemiology and definition.
**Herraiz et al. [22]**	2007	Claves diagnósticas en los somatosonidos o acúfenos pulsátiles	Review article	Patients with PT	Concepts of PT, diagnostic algorithm and different etiologies.
**McFerran et al. [9]**	2007	Tinnitus	Narrative review	Patients with chronic idiopathic non-pulsatile tinnitus	Concepts of tinnitus (pulsatile or not): epidemiology, central pathophysiology, and management strategies.
**Sonmez et al. [3]**	2007	Imaging of pulsatile tinnitus: a review of 74 patients	Original research (retrospective study)	Patients with PT	Concepts of pulsatile and non-pulsatile tinnitus. Prevalence of different etiologies of PT.
**Meckel et al. [45]**	2007	MR angiography of dural arteriovenous fistulas: diagnosis and follow-up after treatment using a time-resolved 3D contrast-enhanced technique.	Original research (retrospective study)	Patients with DAVF	Time-resolved 3D contrast-enhanced MRA reliably detects and grades DAVFs, and is suitable for post-treatment follow-up.
**Mattox et al. [67]**	2008	Algorithm for evaluation of pulsatile tinnitus.	Original research (retrospective study)	Patients with PT	Prevalence of venous and arterial anomalies in PT patients.
**Madani et al. [28]**	2009	Imaging in Pulsatile Tinnitus	Review article	Patients with PT	Structural and vascular causes of PT.
**Farb et al. [46]**	2009	Cranial Dural Arteriovenous Fistula: Diagnosis and Classification with Time-Resolved MR Angiography	Original research (retrospective study)	Patients suspected of having DAVFs	Time-resolved MRA at 3T shows high diagnostic agreement with DSA in detecting and classifying DAVFs.

**Zipfel et al. [43]**	2009	Cranial dural arteriovenous fistulas: modification of angiographic classification scales based on new natural history data.	Review article (and proposal)	Patients with intracranial DAVFs	Concepts of DAVFs: definition and location.
**Vattoth et al. [5]**	2010	A compartment-based approach for the imaging evaluation of tinnitus	Review article	Patients with tinnitus (pulsatile and non-pulsatile)	Concepts of pulsatile and non-pulsatile tinnitus. Prevalence and definition of different etiologies of PT.
**Brouwer et al. [48]**	2010	Dynamic 320-Section CT Angiography in Cranial Arteriovenous Shunting Lesions	Original research (prospective study)	Patients with AVMs or DAVFs	4D-CTA is a promising non-invasive tool for diagnosing and classifying cranial arteriovenous shunting lesions.
**Willems et al. [50]**	2011	Detection and classification of cranial dural arteriovenous fistulas using 4D-CT angiography: initial experience.	Original research (prospective study)	Patients with cranial DAVFs	4D-CTA detects and classifies most DAVFs accurately, offering a viable noninvasive alternative to digital subtraction angiography.
**Narvid et al. [47]**	2011	CT angiography as a screening tool for dural arteriovenous fistula in patients with pulsatile tinnitus: feasibility and test characteristics.	Original research (prospective study)	Patients with PT	Multidetector CTA shows high sensitivity and specificity in detecting DAVFs, supporting its role as a non-invasive screening tool. Signs that may indicate the presence of a DAVF in CTA.
**Sismanis et al. [27]**	2011	Pulsatile tinnitus: contemporary assessment and management.	Narrative review	Patients with PT	PT: diagnostic algorithm, causes, and treatment strategies.
**Gandhi et al. [39]**	2012	Intracranial Dural Arteriovenous Fistulas: Classification, Imaging Findings, and Treatment	Review article	Patients with intracranial DAVFs	DAVF: classification, imaging modalities, and endovascular treatments, highlighting risk stratification based on venous drainage patterns.
**Hofmann et al. [32]**	2013	Pulsatile tinnitus: imaging and differential diagnosis.	Review article	Patients with PT	Comprehensive classification and imaging-based diagnostic approach to arterial, venous, and arteriovenous causes of PT.
**Fujiwara et al. [51]**	2013	Whole-brain CT Digital Subtraction Angiography of Cerebral Dural AVF using 320-detector CT	Original article (retrospective study)	Patients with cerebral DAVFs	320-detector CT DSA offers a reliable non-invasive alternative to conventional angiography in detecting DAVFs.
**Miller et al. [40]**	2015	Intracranial dural arteriovenous fistulae: clinical presentation and management strategies.	Review article	Patients with intracranial DAVFs	DAVF: classification, clinical manifestations, imaging features, and treatment options.
**Bae SC et al. [26]**	2015	Single-Center 10-Year Experience in Treating Patients	Original research (retrospective study)	Vascular tinnitus	Diagnostic Approaches and Treatment Outcomes of PT. Diagnostic work up and principal etiologies.

		With Vascular Tinnitus: Diagnostic Approaches and Treatment Outcomes			
**Miller et al. [10]**	2016	Arterial abnormalities leading to tinnitus	Review article	Patients with arterial causes of PT	Pathophysiology of PT. Arterial sources, imaging protocols and treatment considerations.
**Raghavan et al. [38]**	2016	Advanced neuroimaging of tinnitus	Review article	Patients with tinnitus	Advanced imaging of DAVF.
**Reardon et al. [61]**	2016	Venous abnormalities leading to tinnitus: Imaging evaluation.	Review article	Patients with PT	Venous anomalies leading to PT, especially IIH and high-riding / dehiscent jugular bulb: imaging diagnostic and treatment.
**Serulle et al. [41]**	2016	Dural arteriovenous fistulae: Imaging and Management.	Review article	Patients with intracranial DAVFs	DAVF: pathophysiology, classification, imaging and therapy.
**Pegge et al. [25]**	2017	Pulsatile tinnitus: differential diagnosis and radiological work-up	Review article	Patients with PT	Radiological imaging pathways and differential diagnoses for PT, emphasizing 4D-CTA as a valuable diagnostic tool.
**Lenck et al. [11]**	2017	Stenting of the lateral sinus in idiopathic intracranial hypertension according to the type of stenosis	Original article (retrospective study)	Patients with IIH	New terminology of tinnitus, not subjective and objective tinnitus.
**Elhammady et al. [42]**	2017	Epidemiology, Clinical Presentation, Diagnostic Evaluation, and Prognosis of Cerebral DAVFs	Book chapter	Patients with DAVF	DAVFs: definition, classification and location.
**Aguilar-Pérez et al. [62]**	2017	Endovascular treatment of idiopathic intracranial hypertension: results in 51 patients	Original article (retrospective study)	Patients with IIH	Endovascular venous sinus stenting appears safe and effective in alleviating symptoms in IIH patients.
**Narsinh et al. [56]**	2022	Management of vascular causes of pulsatile tinnitus.	Narrative review	Patients with vascular causes of PT	Treatment strategies for vascular PT.
**Narsinh et al. [24]**	2022	Diagnostic approach to pulsatile tinnitus: a narrative review.	Narrative review.	Patients with pulsatile tinnitus	Pathophysiology, diagnostic protocol and different etiologies of PT.
**Chen et al. [58]**	2023	Expanding the clinical spectrum of idiopathic intracranial hypertension.	Narrative review	Patients with IIH	IIH: epidemiology, clinical manifestations and radiological signs.
**Houdart et al. [12]**	2023	Acúfenos objetivos	Narrative review	Patients with PT and rhythmic tinnitus	Concept, pathophysiology, diagnostic algorithm and different etiologies of PT,
**Korsbaek et al. [57]**	2023	Diagnosis of idiopathic intracranial hypertension: A proposal for evidence-based diagnostic criteria.	Original research (prospective study)	Patients with suspected IIH	Diagnostic criteria for IIH using neuroimaging signs, papilledema, and CSF pressure.

**Wang et al. [26]**	2024	Pulsatile tinnitus: Differential diagnosis and approach to management.	Narrative review	Patients with PT	Diagnostic work-up and management of PT. Different etiologies of PT.
**Friedman et al. [60]**	2024	The Pseudotumor Cerebri Syndrome	Clinical review	Adults and children with IIH/PTCS	Classification of PTCS
**Daou BJ et al. [66]**	2024	Causes of Pulsatile Tinnitus and Treatment Options	Clinical review	Patients with PT	Diagnostic work-up and management of PT. Especially venous etiologies.

*PT: Pulsatile Tinnitus; MR: Magnetic Resonance; DAVF: Dural Arteriovenous Fistula; MRA: Magnetic Resonance Angiography; DSA: Digital Subtraction Angiography; AVM: Arteriovenous Malformation; CT: Computed-Tomography; IIH: Idiopathic Intracranial Hypertension; CSF: cerebrospinal fluid; PTCS: Pseudotumor Cerebri Syndrome.
